# Prevalence of Tinnitus in Community-Dwelling Japanese Adults

**DOI:** 10.2188/jea.JE20100124

**Published:** 2011-07-05

**Authors:** Kaori Fujii, Chisato Nagata, Kozue Nakamura, Toshiaki Kawachi, Naoyoshi Takatsuka, Shino Oba, Hiroyuki Shimizu

**Affiliations:** 1 Department of Epidemiology & Preventive Medicine, Gifu University Graduate School of Medicine, Gifu, Japan; 2 Department of Prevention for Lifestyle-Related Diseases, Gifu University Graduate School of Medicine, Gifu, Japan; 3 Sakihai Institute, Gifu, Japan

**Keywords:** tinnitus, prevalence, population-based, Japanese

## Abstract

**Background:**

Several studies have reported the prevalence of tinnitus among general populations; however, most of these studies were conducted in Europe or the United States. We estimated the prevalence of tinnitus among the general adult population in Japan.

**Methods:**

The subjects were participants in the Takayama Study, a population-based cohort study. In 2002, a total of 14 423 adults (6450 men and 7973 women) aged 45 to 79 years responded to a self-administered questionnaire that inquired about history of tinnitus, which was defined as episodes lasting longer than 5 minutes, excluding those occurring immediately after noise exposure. Respondents were also asked about the loudness and severity of tinnitus.

**Results:**

Overall, 11.9% of the subjects reported having tinnitus; the percentage was somewhat higher among men (13.2%) than women (10.8%). The prevalence of tinnitus increased with age in both sexes. Approximately 0.4% of the overall population reported that tinnitus had a severe effect on their ability to lead a normal life. Medical history of hypertension or ischemic heart diseases, use of steroid or antihypertensive medication, and employment as a factory worker or machine operator were associated with tinnitus status in both men and women.

**Conclusions:**

Tinnitus is relatively common in Japan. Although the use of various definitions of tinnitus in different studies makes it difficult to compare prevalence among populations, the present prevalence estimate was similar to those in studies in Europe and the United States.

## INTRODUCTION

Tinnitus is defined as sound perceived in the ear or head for which there is no acoustic source external to the head. Thus, tinnitus is an entirely subjective experience that can only be described by patient reports.^1^ Tinnitus prevalence has been reported in several studies of general populations.^2^^–^^12^ These studies indicate that approximately 10% to 15% of the population experience tinnitus.^11^ However, most of these studies were conducted among populations in Europe and the United States. In Japan, only 1 study to date has examined the prevalence of tinnitus among the general population.^12^ However, the study subjects were limited to elderly adults: the prevalence of tinnitus was 18.6% among 1320 residents of a community in Japan who were aged 65 years or older. In that study, participants were asked to report any history of tinnitus in the past year. The Comprehensive Survey of Living Conditions in Japan investigated the prevalence of tinnitus in the population.^13^ In that survey, participants were asked whether they had felt unwell or uncomfortable due to ill health or injury in the past few days. If they answered affirmatively, they were asked to indicate the symptoms they had experienced from a list of 42 general symptoms, including tinnitus. According to the 2007 survey, tinnitus was reported by 3.8% of the entire adult population. The percentage among those aged 65 years or older was 7.4%. Thus, the prevalence of tinnitus appears to vary with the definition used. In the present study, we used the definition of tinnitus employed in previous studies to determine its prevalence among adults living in a community in Japan. We also attempted to identify the factors associated with tinnitus status.

## METHODS

This survey was conducted among participants of the Takayama Study, which is a population-based cohort study initiated in 1992. The design and methodology of that study has been described elsewhere.^14^^,^^15^ All nonhospitalized residents aged 35 years or older in Takayama City, Gifu were invited to participate in the study. A total of 31 552 persons, yielding a participation rate of 85.3%, completed a baseline questionnaire that included questions on demographic characteristics, smoking and drinking habits, diet, exercise, occupation, and medical and reproductive histories. In July 2002, we sent a questionnaire that inquired about history of tinnitus to those who were younger than 70 years of age at the baseline in 1992. We obtained residential registry data on death and moves between September 1992 and March 2000, but not after that period. Among 22 435 individuals, after the exclusion of those known to have died or moved, 14 975 responded to the questionnaire. The details are described elsewhere.^16^ The response rate was 66.7%.

To identify tinnitus cases, the following question was asked: “Have you ever had tinnitus lasting longer than 5 minutes? Do not include when this happened immediately after very loud sounds.” This was the criterion proposed by Coles.^3^ Each participant was asked to choose from 3 responses: “I have never had tinnitus,” “I have tinnitus,” or “I have had tinnitus.” Those individuals reporting a history of tinnitus were asked to provide information concerning loudness, severity, and age at onset. Tinnitus severity was assessed by the answers to 2 questions, one on the regularity of tinnitus during waking hours and the other on the extent to which tinnitus affected the respondent’s ability to lead a normal life. These questions were selected from questionnaires developed by Coles^3^ and Erlandsson.^17^ We also asked participants to provide demographic information and details of their smoking status, exercise, and medical histories. Exercise was assessed by asking respondents about the average hours per week spent performing various kinds of activities during the past year. The activities were categorized into strenuous sports (jogging, bicycling on hills, tennis, racquetball, swimming, laps, or aerobics), vigorous work (moving heavy furniture, loading or unloading trucks, shoveling, weight lifting, or equivalent manual labor), and moderate activity (brisk walking, golfing, bowling, bicycling on level ground, or gardening). The time per week spent at each intensity was multiplied by its corresponding energy expenditure requirement, expressed in metabolic equivalent (METs), and summed to yield a MET score (METs·h/week). The details, including the analysis of the validity of the method, are described elsewhere.^18^ Informed consent was obtained from each subject, and the study was approved by the ethical board of the Gifu University Graduate School of Medicine.

A total of 552 subjects (224 men and 328 women) did not answer the questions about tinnitus. The remaining 14 423 (6450 men and 7973 women) were included in the present analysis. After taking into account the participation rate (85.3%) in the Takayama Study, the ultimate response rate to the tinnitus questionnaire was approximately 55%.

The prevalence of tinnitus was calculated according to age and sex. Logistic regression models were used to examine associations of tinnitus with marital status, years of education, body size, smoking and drinking habits, exercise, and occupational and medical histories. Information about alcohol intake and occupation was obtained at baseline only. To evaluate the effect of nonresponse on the estimation of prevalence, we examined baseline data from the 2002 questionnaire to determine whether variables shown to be associated with tinnitus differed between respondents and nonrespondents.

## RESULTS

The tinnitus status of the respondents is shown in Table 1. Overall, 11.9% of subjects reported that they had recurrent tinnitus. The percentage was somewhat higher among men (13.2%) than women (10.7%). The percentage reporting a history of tinnitus was 16.0%. The prevalence of tinnitus increased with age in both sexes. The peak was at age 70 to 79 years: 15.5% of men and 14.5% of women in this age group reported tinnitus.

**Table 1.** Prevalence of tinnitus

Age (years)	Men							Women						
	
	Never		Past		Current		Total	Never		Past		Current		Total
					
	*n*	%	*n*	%	*n*	%		*n*	%	*n*	%	*n*	%	
45–49	528	88.3	14	2.3	56	9.4	598	767	92.0	26	3.1	41	4.9	834
50–59	1743	86.7	51	2.5	217	10.8	2011	2262	87.0	115	4.4	222	8.6	2599
60–69	1822	82.1	69	3.1	329	14.8	2220	2204	82.0	158	5.9	326	12.1	2688
70–79	1306	80.6	64	3.9	251	15.5	1621	1479	79.8	105	5.7	268	14.5	1852
Total	5399	83.7	198	3.1	853	13.2	6450	6712	84.2	404	5.1	857	10.7	7973

Table 2 shows the loudness distribution, regularity of tinnitus during waking hours, and extent of the inability to lead a normal life among those reporting current tinnitus. Approximately 50% reported low levels of sound associated with tinnitus. However, 20% to 30% reported an awareness of tinnitus during waking hours. Approximately 0.4% of the total population reported that tinnitus severely affected their ability to lead a normal life.

**Table 2.** Loudness, regularity, and severity of tinnitus among those reporting current tinnitus

Age (years)	45–49		50–59		60–69		70–79		total	
				
	No.	%	No.	%	No.	%	No.	%	No.	%
Men										
Loudness										
Low	39	69.6	113	52.1	147	44.7	123	49.0	422	49.5
Moderate	12	21.4	70	32.2	120	36.5	75	29.9	277	32.5
Loud	4	7.2	22	10.1	39	11.8	25	9.9	90	10.5
Very loud	1	1.8	6	2.8	9	2.7	11	4.4	27	3.2
Unknown	0	0.0	6	2.8	14	4.3	17	6.8	37	4.3
Regularity										
Rarely	29	51.8	80	36.9	113	34.3	79	31.5	301	35.3
Sometimes	8	14.3	28	12.9	34	10.3	35	13.9	105	12.3
Usually	10	17.8	53	24.4	63	19.2	46	18.3	172	20.1
Always	8	14.3	51	23.5	105	31.9	72	28.7	236	27.7
Unknown	1	1.8	5	2.3	14	4.3	19	7.6	39	4.6
Annoyance										
Not at all	35	62.5	101	46.6	151	45.9	119	47.4	406	47.6
Slight	14	25.0	58	26.7	95	28.9	59	23.5	226	26.5
Moderate	5	8.9	46	21.2	56	17.0	55	21.9	162	19.0
Severe	1	1.8	4	1.8	12	3.6	8	3.2	25	2.9
Unknown	1	1.8	8	3.7	15	4.6	10	4.0	34	4.0

Women										
Loudness										
Low	29	70.7	125	56.3	174	53.4	131	48.9	459	53.6
Moderate	6	14.6	69	31.1	87	26.7	74	27.6	236	27.5
Loud	4	9.8	19	8.6	30	9.2	22	8.2	75	8.7
Very loud	0	0.0	2	0.9	3	0.9	7	2.6	12	1.4
Unknown	2	4.9	7	3.1	32	9.8	34	12.7	75	8.8
Regularity										
Rarely	23	56.1	104	46.8	131	40.2	99	36.9	357	41.7
Sometimes	9	21.9	25	11.3	38	11.6	33	12.3	105	12.2
Usually	4	9.8	43	19.4	53	16.3	36	13.4	136	15.9
Always	4	9.8	38	17.1	76	23.3	72	26.9	190	22.2
Unknown	1	2.4	12	5.4	28	8.6	28	10.5	69	8.0
Annoyance										
Not at all	24	58.5	106	47.7	162	49.7	113	42.2	405	47.3
Slight	9	22.0	69	31.1	83	25.4	75	28.0	236	27.5
Moderate	7	17.1	34	15.3	55	16.9	50	18.6	146	17.0
Severe	0	0.0	3	1.4	10	3.1	14	5.2	27	3.2
Unknown	1	2.4	10	4.5	16	4.9	16	6.0	43	5.0

Table 3 shows the age-adjusted ORs of tinnitus according to sociodemographic and clinical factors. Years of education, exercise, and some occupational and medical factors were significantly associated with tinnitus status. Among the factors that were significantly associated with tinnitus, years of education, exercise, and occupational history of mining/quarrying/rock crushing/cement manufacturing significantly differed among male respondents and nonrespondents after controlling for age; in comparison with respondents, nonrespondents were less likely to have attained a high level (≥15 years) of education (11.7% vs 11.1%, respectively) and less likely to been exercisers (67.7% vs 65.3% for ≥7.5 METs·h/wk). They were more likely to be have been engaged in mining/quarrying/rock crushing/cement manufacturing (1.6% vs 2.1%). In women, the distributions of factors associated with tinnitus were similar between respondents and nonrespondents. The details of the baseline characteristics of nonrespondents and respondents have been described elsewhere.^15^

**Table 3.** Age-adjusted odds ratios (ORs) and 95% confidential intervals (CIs) for tinnitus according to sociodemographic and clinical factors

	Men		Women	
		
	OR	95% CI	OR	95% CI
Marital status				
Married	1.00		1.00	
Not married	1.25	(0.97–1.61)	1.08	(0.91–1.28)
Education, years				
≤11	1.00		1.00	
12–14	1.01	(0.85–1.19)	0.89	(0.75–1.06)
≥15	0.76	(0.58–0.99)	0.76	(0.50–1.14)
BMI (kg/m^2^)				
<20	1.00		1.00	
20–22	0.99	(0.79–1.25)	0.83	(0.67–1.02)
22.1–24	0.98	(0.78–1.22)	1.01	(0.83–1.23)
>24	1.02	(0.82–1.28)	0.90	(0.73–1.11)
Smoking				
Never smokers	1.00		1.00	
Ex-smokers	0.86	(0.71–1.04)	1.08	(0.84–1.38)
Current smokers	1.02	(0.83–1.25)	1.36	(0.94–1.97)
Alcohol intake^a^				
0	1.00		1.00	
Low	1.12	(0.82–1.53)	1.00	(0.83–1.19)
High	1.04	(0.76–1.42)	1.00	(0.84–1.20)
Exercise (METs·h/wk)				
0–7.4	1.00		1.00	
7.5–25.4	1.27	(1.06–1.53)	1.13	(0.95–1.35)
25.5–	1.25	(1.04–1.51)	0.95	(0.80–1.14)
Occupational history ​ (yes/no)				
Laborer or farm worker^b^	0.97	(0.77–1.21)	1.14	(0.86–1.51)
Factory worker ​ or machine operator^b^	1.31	(1.07–1.60)	1.34	(1.01–1.78)
Clerical or office worker^b^	0.89	(0.70–1.14)	0.99	(0.79–1.25)
Sales^b^	1.00	(0.75–1.32)	1.07	(0.71–1.62)
Manager or administrator^b^	1.03	(0.81–1.33)	2.00	(0.87–4.62)
Small business owner^b^	0.64	(0.42–0.96)	0.75	(0.50–1.11)
Professional/technical^b^	1.12	(0.94–1.34)	1.11	(0.84–1.47)
Metal production or ​ processing^c^	1.52	(1.06–2.19)	1.21	(0.42–3.46)
Mining quarrying, rock ​ crushing, or ​ cement manufacturing^c^	1.98	(1.26–3.14)	2.46	(0.50–12.02)
Cotton, wool, or textile ​ processing^c^	1.41	(0.68–2.91)	1.91	(1.27–2.87)
Plastic, pesticide, ​ or paint production/ ​ gasoline refining^c^	1.12	(0.75–1.67)	1.49	(0.78–2.85)
Chemical work^c^	0.80	(0.28–2.26)	6.57	(2.60–16.55)
Furniture making or ​ woodworking^c^	1.10	(0.89–1.36)	1.25	(0.94–1.66)
Automotive repair^c^	1.31	(0.90–1.89)	0.95	(0.29–3.13)
Medical history (yes/no)				
Hypertension	1.36	(1.17–1.60)	1.52	(1.29–1.79)
Diabetes	1.11	(0.88–1.40)	0.95	(0.67–1.35)
Ischemic heart disease	1.29	(1.00–1.65)	1.66	(1.27–2.17)
Stroke	0.73	(0.44–1.23)	1.01	(0.53–1.90)
Cancer	1.15	(0.81–1.62)	1.32	(0.96–1.83)
Asthma	1.14	(0.81–1.59)	1.78	(1.32–2.41)
Medication use				
Antihypertensive	1.44	(1.20–1.72)	1.57	(1.30–1.89)
Steroid	1.57	(1.11–2.21)	2.46	(1.92–3.13)

^a^The cut-offs between low and high intake categories are 38.5 ml/day or more in men and 3.7 ml/day or more in women.

^b^Indicates industry of longest employment.

^c^Employed for ≥10 years.

## DISCUSSION

The overall prevalence of tinnitus in adults aged between 45 and 79 years was 11.9%. Although only 0.4% of adults reported that tinnitus severely affected their ability to lead a normal life, this finding should still be regarded as important because of the poor results of tinnitus treatment.^19^

The prevalence of tinnitus in the general adult population has been estimated in a number of countries (Table 4). In a study conducted in Japan,^12^ tinnitus was present in 15.3% and 18.9% of adults aged 65 to 69 years and 70 to 79 years, respectively. Prevalence in the corresponding age groups was somewhat lower in the present study. However, a much lower prevalence (5.2% for those aged 45–79 years) was found in the Comprehensive Survey of Living Conditions in Japan,^13^ in which only individuals who indicated that they felt unwell or uncomfortable were asked about their experience of tinnitus. Thus, cases of tinnitus that occurred infrequently or were considered unimportant by a survey participant might have been excluded. Differences in individual perception of illness among participants may have affected the results of the survey. The British National Study of Hearing, which started in 1978,^3^ reported that approximately 15% of adults aged 17 years or older had tinnitus persisting longer than 5 minutes, after excluding tinnitus occurring immediately after exposure to noise (prolonged spontaneous tinnitus). Tinnitus severely reduced the ability to lead a normal life in 0.5% of adults. Because 2 other studies^4^^,^^8^ had used the same definition for tinnitus, we used the same phrasing for the questions in the present study. Unfortunately, different definitions have been used in other studies. Because differences in tinnitus prevalence among studies are likely to depend on subject age and the definition of tinnitus, its prevalence did not vary greatly among populations. Studies found that tinnitus prevalence increased with age, although there was a plateau at either 60 to 69 years^4^^,^^9^^,^^10^ or 70 to 79 years,^5^^,^^11^ with a subsequent decline in older age groups. A somewhat higher prevalence among men than women has been reported in some^2^^,^^7^^,^^11^^,^^12^ but not all studies.^5^^,^^6^^,^^9^^,^^10^

**Table 4.** Previous studies reporting prevalence of tinnitus in a general population

Authors	Year	Study subjects	Tinnitus definition	Prevalence
Leske^2^	1981	National Health Examination Survey of 1960–62, USA 6672 persons aged 18–79 years	Tinnitus during the few years before the study	32%
Coles^3^	1984	National Study of Hearing, Pilot study, UK 5000 persons aged 17 years or older	Prolonged spontaneous tinnitus^a^	15.5%–18.6%
Davis^4^	1989	National Study of Hearing, UK 35 330 persons aged 17 years or older	Prolonged spontaneous tinnitus	9.7%
Axelsson & Ringdahl^5^	1989	Sweden 2378 persons aged 20–80 years	Tinnitus often or always present	14.2%
Gates et al^6^	1990	Framingham cohort, USA 5209 men who were free of CVD aged 60 years or older	Current tinnitus	16.8%
Cooper^7^	1994	Health and Nutrition Examination Survey of 1971–75, USA 6342 persons aged 25–74 years	Frequent, bothersome tinnitus over the past few years	14.9%
Quaranta et al^8^	1996	Italy 2170 persons aged 18 years or older	Prolonged spontaneous tinnitus	14.5%
Nondahl et al^9^	2002	Epidemiology of Hearing Loss Study, USA 3753 persons aged 48–92 years	Tinnitus of at least moderate severity or causing difficulty in falling asleep in the past year	8.2%
Sindhusake et al^10^	2003	Blue Mountains Hearing Study, Australia 2015 persons aged 55–99 years	Prolonged tinnitus	30.3%
Hoffman & Read^11^	2004	National Health Interview Survey of 1990 and 1994–95, USA 59 343 persons aged 20 years or older (1990)	Tinnitus in the previous 12 months	8.4%
		99 435 persons aged 20 years or older (1994–95)	Tinnitus persisting at least 3 months	4.4%
Michikawa et al^12^	2010	Kurabuchi Study, Japan 1320 persons aged 65 years or older	Tinnitus in the past year	18.6%

^a^Tinnitus persisting longer than 5 minutes, excluding that occurring immediately after noise exposure.

High noise levels can cause hair cell damage.^20^ Work-related exposure to noise has been reported to be associated with tinnitus,^8^^,^^21^ and we confirmed that workers exposed to occupational noise were more likely to have tinnitus. An association between history of cardiovascular disease and tinnitus has been reported in several studies.^8^^,^^9^^,^^12^ The mechanism for this association is unclear, but the function of the auditory nervous system may be affected by cardiovascular disease.^19^^,^^22^ The observed associations of tinnitus with histories of ischemic heart disease and hypertension provide additional evidence that cardiovascular disorders may be a potential risk factor of tinnitus. Numerous drugs and chemicals, including antimicrobials, diuretics, antineoplastic drugs, and salicylates and other nonsteroidal anti-inflammatory drugs, have been reported to be potentially ototoxic.^23^ A history of asthma and use of steroids were associated with tinnitus in the present study. Because asthma involves inflammatory mediators common in the pathogenesis of cardiovascular disease,^24^ it is possible that asthma causes tinnitus. Steroids are not classified as ototoxic medications. Thus, it is not steroid use itself, but rather the clinical indications for steroid administration, including asthma, that might play a role in tinnitus. Furthermore, the use of steroids as a treatment for tinnitus or hearing loss, which often accompanies tinnitus,^20^ may be another explanation for the observed association between steroid use and tinnitus.

The risk factors for tinnitus are not well known. Many theories have been proposed to explain the pathophysiologic basis of tinnitus,^20^ and there are likely to be many possible mechanisms, due to the potential effects of previous disease history and medication use on tinnitus. Although exercise was associated with tinnitus in men, there is no plausible biological explanation for exercise as a cause of tinnitus. Because of the cross-sectional nature of the present data, no causal inferences can be drawn regarding observed associations. Perhaps a diagnosis of tinnitus or experience of related symptoms prompted some men to begin exercise programs.

Because we could not thoroughly identify decedents due to lack of information in the city registry, the response rate in the present study might be underestimated. However, it is unlikely that such underestimation was large. Therefore, we should consider the possibility that nonresponse bias affected the results. It was an advantage that we were able to compare the characteristics of respondents and nonrespondents using information from a baseline questionnaire of this cohort. The observed associations of tinnitus with level of education and mining/quarrying/rock crushing/cement manufacturing suggest that we might have underestimated prevalence among men. However, the association of exercise with tinnitus suggests an overestimation of prevalence. Nonetheless, the magnitude of these associations and the distributions of the variables in respondents and nonrespondents suggests that the effect of nonresponse bias was not large. It must be kept in mind, however, that the comparison of the baseline characteristics of nonrespondents and respondents may or may not be valid, depending on changes in these variables after the baseline survey. Self-reporting of tinnitus is another limitation of the study. However, because tinnitus is by its nature subjective, there is no objective measure to prove its existence or verify reported severity.

In conclusion, we found that 11.9% of community-dwelling Japanese adults aged 45 to 79 years had tinnitus, and approximately 0.4% experienced tinnitus that was severe enough to reduce their ability to lead a normal life. Although the differing definitions of tinnitus used in studies make it difficult to compare prevalence among populations, the present prevalence study was similar to estimates in studies of European and US populations. Further studies should be encouraged to thoroughly identify the causes, characteristics, and impact of tinnitus in affected individuals.

## References

[1] Meikle MB A conceptual framework to aid the diagnosis and treatment of severe tinnitus . Aust N Z J Audiol. 2002;24:59–67 10.1375/audi.24.2.59.31107

[2] Leske MC Prevalence estimates of communicative disorders in the U.S. language, hearing and vestibular disorders . ASHA. 1981;23:229–37 6972221

[3] Coles RR Epidemiology of tinnitus: (1) prevalence . J Laryngol Otol Suppl. 1984;9:7–15 6596362 10.1017/s1755146300090041

[4] Davis AC The prevalence of hearing impairment and reported hearing disability among adults in Great Britain . Int J Epidemiol. 1989;18:911–7 10.1093/ije/18.4.911 2621028

[5] Axelsson A , Ringdahl A Tinnitus—a study of its prevalence and characteristics . Br J Audiol. 1989;23:53–62 10.3109/03005368909077819 2784987

[6] Gates GA , Cooper JC Jr , Kannel WB , Miller NJ Hearing in the elderly: The Framingham cohort, 1983–1985. Part I. Basic audiometric test results . Ear Hear. 1990;11:247–56 10.1097/00003446-199008000-00001 2210098

[7] Cooper JC Jr Health and nutrition examination survey of 1971–75: Pert II. Tinnitus, subjective hearing loss, and well-being . J Am Acad Audiol. 1994;5:37–43 8155893

[8] Quaranta A , Assennato G , Sallustio V Epidemiology of hearing problems among adult in Italy . Scand Audiol Suppl. 1996;42:9–13 8668911

[9] Nondahl DM , Cruickshanks KJ , Wiley TL , Klein R , Klein BE , Tweed TS Prevalence and 5-years incidence of tinnitus among older adults: The epidemiology of hearing loss study . J Am Acad Audiol. 2002;13:323–31 12141389

[10] Sindhusake D , Mitchell P , Newall P , Golding M , Rochtchina E , Rubin G Prevalence and characteristics of tinnitus in older adults: the blue mountains hearing study . Int J Audiol. 2003;42:289–94 10.3109/14992020309078348 12916702

[11] Hoffman HJ, Reed GW. Epidemiology of tinnitus. In: Snow JB Jr, editor. Tinnitus: Theory and Management. Lewiston, NY; 2004. p. 16–41.

[12] Michikawa T , Nishiwaki Y , Kikuchi Y , Saito H , Mizutari K , Okamoto M , Prevalence and factors associated with tinnitus: A community-based study of Japanese elders . J Epidemiol. 2010;20:271–6 10.2188/jea.JE20090121 20501961 PMC3900786

[13] Statistics and Information Department, Minister’s Secretariat, Ministry of Health, Labour and Welfare. Comprehensive Survey of Living Conditions. Health and Welfare Statistics Association; 2009.

[14] Shimizu H. The Basic Report on Takayama Study. Gifu, Japan: Department of Public Health, Gifu University School of Medicine; 1996.

[15] Nagata C , Takatsuka N , Shimizu H Soy and fish oil intake and mortality in a Japanese community . Am J Epidemiol. 2002;156:824–31 10.1093/aje/kwf118 12397000

[16] Nagata C , Nakamura K , Fujii K , Kawachi T , Takatsuka N , Oba S , Smoking and risk of cedar pollinosis in Japanese men and women . Int Arch Allergy Immunol. 2008;147:117–24 10.1159/000135698 18520156

[17] Erlandsson SI , Hallberg LR , Axelsson A Psychological and audiological correlates of preceived tinnitus severity . Audiology. 1992;31:168–79 10.3109/00206099209072912 1642568

[18] Suzuki I , Kawakami N , Shimizu H Reliability and validity of a questionnaire for physical activity in epidemiological studies . J Epidemiol. 1998;8:152–9 Supplementary comment: J Epidemiol. 2002;12:54 9782671 10.2188/jea.8.152

[19] Patterson MB , Balough BJ Review of pharmacological therapy for tinnitus . Int Tinnitus J. 2006;12:149–59 17260881

[20] Henry JA , Dennis KC , Schechter MA General review of tinnitus: prevalence, mechanisms, effects, and management . J Speech Lang Hear Res. 2005;48:1204–35 10.1044/1092-4388(2005/084) 16411806

[21] Sindhusake D , Golding M , Newall P , Rubin G , Jakobsen K , Mitchell P Risk factors for tinnitus in apopulation of older adults: The Blue Mountain Hearing Study . Ear Hear. 2003;24:501–7 10.1097/01.AUD.0000100204.08771.3D 14663349

[22] Møller AR Tinnitus: presence and future . Prog Brain Res. 2007;166:3–16 10.1016/S0079-6123(07)66001-4 17956767

[23] Seligmann H , Podoshin L , Ben-David J , Fradis M , Goldsher M Drug-induced tinnitus and other hearing disorders . Drug Saf. 1996;14:198–212 10.2165/00002018-199614030-00006 8934581

[24] Appleton SL , Ruffen RE , Wilson DH , Tayloe AW , Adams RJ Asthma is associated with cardiovascular disease in a representative population sample . Obes Res Clin Pract. 2008;2:91–9 10.1016/j.orcp.2008.04.005 24351727

